# Personnel shortages and the provision of long-term care: an empirical analysis of German nursing homes

**DOI:** 10.1007/s10198-025-01782-7

**Published:** 2025-05-09

**Authors:** Dörte Heger, Annika Herr, Maximilian Lückemann, Arndt Reichert, Leonie Tycher

**Affiliations:** 1 RWI- Leibniz Institute for Economic Research, Essen, Germany; 2 Hochschule Bochum, Gesundheitscampus 6-8, Bochum, 44801 Germany; 3 Institute of Health Economics, Leibniz University Hannover, Königsworther Platz 1, Hannover, 30167 Germany

**Keywords:** Nursing homes, Personnel shortage, Capacity, C90, I10, I11, I18, J01

## Abstract

Amidst demographic shifts, advanced economies are facing critical nursing shortages. This study analyzes how long-term care in German nursing homes relate to these shortages using administrative data for the period 2007 to 2017. Our analysis reveals that higher nursing shortages correlate with decreased nurse-to-resident ratios, changes in the qualification mix of nurse personnel, and reduced occupancy rates. These findings suggest that nurse shortages might be a threat to the quality of care and the financial sustainability of nursing homes at the margin of being profitable.

## Introduction

In numerous advanced economies, there has been a growing discrepancy between the number of retiring workers and incoming younger entrants in the labor market. Such demographic shifts have significantly contributed to labor shortages in various sectors [e.g., 1]. A prime example is the nursing care sector, where personnel shortages have been escalating over time, reaching critical levels [2, 3].

Important health care consequences of nurse shortages likely include prolonged waiting lists for people in need of care, inadequate care, and in extreme cases, the shutdown of entire nursing homes [4, 5]. To make matters worse, the same demographic shift also generates an increased demand of nursing care. In certain countries, like Germany, the magnitude of the problem has become so glaring that it has spurred intense public debates. These discussions recently started to revolve around triage protocols, questioning whether nursing homes should deprioritize elderly individuals with minimal care needs [for an example from German media, see 6].

In this paper, we aim to investigate the consequences of labor shortages within the elderly care sector in Germany for long-term care. We employ data from the official German Care Statistic spanning 2007 to 2017, which encompasses the universe of German care facilities, personnel, and care recipients (both formal and informal). This data set is augmented with administrative vacancy information at the county level on all publicly reported nursing positions that stem from the capacity planning of individual nursing homes, provided by the Research Institute of the Federal Employment Agency [7].

Nursing homes facing nurse shortages are compelled to implement traditional capacity planning measures, such as job advertisements in local newspapers and at the Federal Employment Agency, to continue operating without turning away residents and, thus, preserving their revenue stream. Once these measures are exhausted, nursing homes might operate with a decreased nurse-to-resident ratio and a higher share of relatively less scarce nurse assistants (NAs). However, these changes can dilute the quality of personalized care that residents receive.^1^ With a shortage of (qualified) nursing staff, a higher risk of compromised safety and a greater likelihood of mistreatment of nursing home residents as well as reduced attention to detail have been reported [9–13]. While recent literature reviews report mostly positive findings for staffing levels for hospitals but mixed results for nursing homes, a positive relationship is generally found between registered nurse (RN) employment and quality outcomes also in nursing homes [see, e.g. 8, 14, 15, 16].

With this risk for the quality of care in mind, regulators often stipulate minimum nurse-to-resident ratios and staff qualification levels, which act as a safeguard to prevent a decline in care quality [17]. As a result, nursing homes having reached the critical threshold are forced to leave rooms empty. In this case, the occupancy rate will decrease, potentially diminishing profitability because nursing homes are required to finance investment costs to a large extent through revenues and nursing home prices are negotiated based on the assumption of maximum utilization of available beds. With persistently reduced occupancy rates, the long-term financial viability of these institutions potentially comes into question. In fact, current trends suggest that every fourth nursing home operates at a deficit [18].

This paper empirically analyzes how various metrics of nursing homes including the nurse-to-resident ratio, the qualification mix of the nursing personnel, and the occupancy rate correlate with the county-level number of published vacancy notes, which we use as our measure of nursing shortages. Notably, on average, there are three times as many vacancy notes as there are nursing candidates, indicating that many of these positions remain vacant for extended periods [19]. Our methodological approach involves utilizing pooled cross-sectional data and linear regression analyses. The examined nursing home metrics are also highly relevant from a public health policy perspective. For instance, the nurse-to-resident ratio likely serves as proxy for the nursing homes’ ability to provide personnel intensive, high-quality care [e.g., 9]. Moreover, the occupancy rate potentially approximates the financial sustainability of nursing homes.

We find higher nursing shortages to be accompanied by a substantially lower nurse-to-resident ratio, a shift of the staff qualification mix, and a reduced overall provision of care. As to the latter, we find a negative relationship between nurse shortages and the number of nursing home residents. We also find that the occupancy rate is reduced. Consequently, the looming nursing shortages stand as a potential threat to the sustainability and future of elderly care in countries like Germany. In our discussion, we draw from existing literature and present potential public policy measures that can be employed by policymakers aimed at addressing nursing personnel shortages [e.g. 20, 21, 22, 23, 24].

This paper is the first to empirically study the consequences of nursing shortages for long-term care provided by nursing homes. In doing so, we provide additional insights into the larger literature studying shortages in the nursing home market. As opposed to nursing shortages, Ching et al. [25] show that limited bed capacity due to entry regulations results in the rationing of services for nursing home residents, leaving care-dependent individuals without a nursing home bed. Other studies estimate the effects of nursing shortages on the well-being of the nursing workforce [e.g., 26]. Harrington and Swan [27] assess the relationship between nursing hours and nurse turnover rates, resident case mix, and nursing wages. In doing so, they develop a better understanding of provider decisions in relation to nurse capacity planning, as opposed to the consequences for nursing home metrics within existing capacity examined in our paper. Our empirical results on changes in nursing home metrics when unfilled vacancies increase is novel.

Additionally, our paper connects with several studies that examine the relationship between demand and capacities in nursing homes. For example, Bae et al. [28] simulate the interaction of nursing capacity and demand, finding that appropriate capacity planning can improve system indicators such as waiting times for beds, occupancy, and quality. Herr et al. [29] report that the regional variation in the utilization of nursing home care strongly depends on the supply of both professional care and informal care. Similarly, Pilny and Stroka [30] examine the relationship between the demand for long-term in-patient care and the regional supply of nursing home beds. Van Gameren and Woittiez [31] relate the demand for higher elderly care provision to access to informal care. Bauer and Stroka [32] examine the potential correlation between women’s labor market participation, educational attainment, and nursing home prices. Several studies examine the determinants of nurses’ labor supply and the attractiveness of the nursing profession [33–38].

There is some more literature available for hospital care related to our focus on personnel shortages. Most closely connected with our study is the analysis of Blegen et al. [39], showing that patient care staffing levels in both intensive care and non-intensive care units decrease as the supply of registered nurses in the surrounding geographic area decreases. On the contrary, Winter et al. [40] find no relationship between nursing vacancies and the nurse-to-patient ratio in German hospitals. The authors argue that the latter can be the result of a deliberate choice by hospitals to, for instance, provide outstanding service quality. Rather than deviating from their preferred nurse-to-patient ratio, these hospitals may focus their adjustments to a lack of qualified nursing personnel in the labor market on how care is organized and the nature of the services provided. Further available studies examine ways to use existing nursing capacity in hospitals more effectively [41, 42] and the effects of nursing shortages on quality of medical care as well as care costs [43–47]. During our study period, educational requirements for nurses working in hospitals differed from that for nurses working in nursing homes, leading to separate nursing labor markets in Germany.^2^ Thus, existing results from the hospital sector may not be completely transferable to the nursing home market.

## Institutional background

Nursing homes (NHs) negotiate with the mandatory long-term care insurance funds as well as the social assistance authorities for the poor the price for nursing care (§85 SGB XI) and the price for food and accommodation (§87 SGB XI), see also §75 SGB XI. The former varies according to the care level of the residents. The mandatory long-term care insurance covers a negotiated fixed amount of the care-related costs for each resident and the remaining amount of the total price (additionally including charges to cover NH investment costs) is paid for by the residents (§84-87a SGB XI). For residents relying on social assistance, the respective authority covers the private copayments (§61a SGB XII).

In some federal states, NHs receive public funds to cover a fraction of the calculated investment costs [18, 48]. The underlying calculations require approval by the social assistance authority (§82 SGB XI). Yet, in the other federal states, NHs also require the same approval if they have residents who receive social assistance (§76a SGB XII). This applies to most, if not all, NHs. When negotiated, the pricing for infrastructure maintenance and other investment costs is generally structured so that, with an assumed occupancy rate of about 95 %, the investment costs covered by residents plus any financing from the federal state fully account for the NH’s overall investment costs [49, 50]. In all cases, the price for investment costs is fully borne by the residents or the social assistance authority. Given the fixed pricing and the requirement to (partially) cover investment costs through revenues, the occupancy rates are closely tied to the financial stability of NHs.

The largest part of the marginal costs is driven by nursing care personnel, wherefore the NH reimbursement scheme produces incentives to operate with minimal nurse-to-resident ratios and qualification levels. In particular, economic theory predicts a reduction in the demand of labor to contain costs when wages rise as a result of nursing shortages (see Appendix for details).^3^ As such, according to this theory, reduced staffing ratios represent an indirect result of nursing shortages when NHs use containment measures in response to increased wages. While there is no specific empirical evidence available for nursing homes, this prediction is consistent with findings of [39] for the hospital sector in the United States.^4^

German NHs, however, are restricted by legally binding predefined nurse-to-resident ratios [54, 55] that vary across care levels and federal states and only allow for short-term deviations (see Table 4 for the year 2017, the last year of our observation period, in the Appendix). These regulated nurse-to-resident ratios may impose a bound on the NHs ability to set nursing levels at the theoretical optimum. When the room for substituting qualified nursing personnel (NP), namely registered nurses (RN), with nursing assistants (NA) and employing fewer nurses per resident is exhausted, NHs will stop admitting new residents, i.e., reduce the occupancy rate or the number of beds. Despite a reduction in the number of (more) costly NP per resident, the financial sustainability of NHs can be threatened when NHs experience a drop in revenues required to finance investments. A reduction in the occupancy rate can be particularly problematic for NHs at risk of exiting the market [18]. Hence, nursing shortages can have important implications for both the quality and quantity of provided care.

## Materials and methods

### Data

We combine data covering the (pre-pandemic) period 2007 to 2017 from three sources. First, we use the German Care Statistic provided by the Statistical Offices of the Länder at the Research Data Center of Hannover [56]. It is collected biennially in December and includes rich information on the universe of German NHs and their residents, home health care providers, and their clients. We restrict our analysis to care provision for the elderly in long-term care facilities. We drop individuals below 65 years of age (around 500,000 individuals) and facilities that are only providing day- and short-term care (15,823 facilities). We exploit information on the NH level, covering the number and care level of their permanent residents as well as the personnel qualification mix, contractual working time, and tasks performed by the staff [56].^5^

Second, to capture staff shortages, we include vacancy data from the Research Institute of the Federal Employment Agency [7]. This data provides us with the number of all open positions (yearly averages) in NHs for NA, RN, and specialists aggregated at the county level [7]. Employers are required by law to report open positions to the federal employment agency (§164 SGB IX). Whereas other research aims to approximate personnel shortage by the nurses’ workload [45], open positions are arguably a more direct measure of personnel shortage. NHs publish job advertisements for one specific nurse type [57] even if they look for more than one. Nursing homes may also post job openings to proactively manage nursing turnover and build a reservoir of qualified candidates, thereby speeding up the hiring process when positions become available. In both scenarios, the advertised vacancies do not appropriately reflect nursing personnel shortage. We argue that such measurement error yields attenuation bias and, hence, conservative point estimates in the regression analyses, especially given that our explanatory variable is measured at the county level such that the measurement error is unlikely to be correlated with metrics of individual nursing homes.^6^

Lastly, we draw indicators of regional and urban development from the Federal Office for Building and Regional Planning ( [58], INKAR). The county-level information relates to the working population, medical infrastructure, wealth, and demographic composition.

The analyses are conducted at the research data center in Hannover. To protect the confidentiality of individual care facilities in the Care Statistic, for certain years, we aggregate smaller counties that lack representation of at least three different NH ownership types. This implies some additional measurement error in our main explanatory variable.

As the first set of outcomes, we analyze possible cost containment measures of NHs. Specifically, we study the NP to NH resident ratio (NP/resident) calculated individually for each facility and year, where the number of personnel is measured in full-time equivalent staff (FTE) and we focus on permanent residents.^7^ With the objective of considering differences in skill-mix, we calculate this ratio for all NP, RNs, and NAs separately. While NP does not include all personnel working in a NH (e.g., excluding physiotherapists or housekeeping) it represents the input most crucial to care provision (Fig. 3 in the Appendix lists the professions included in the definitions).

We use the qualification mix as additional cost containment measure that allows us to examine the different compositions of NP and their potential for substitution when relative wages change. For this purpose, we calculate the share of NA among all NP. Specifically, we employ the relation of nursing assistants in FTE to the total number of NP in FTE.

In addition, in order to reflect the possibility that NHs stop admitting new residents and the implications thereof, we utilize the number of beds for permanent care, the number of residents per NH, and the home-specific occupancy rate. This ratio is calculated as the proportion of permanent NH beds occupied by NH residents compared to all available permanent beds in the NH at a given point in time.

We employ the county-specific NP vacancy ratios to assess NP shortage. This measure has already been applied in other studies [59–61]. Here, we define the vacancy ratio (NP-vacancy) as the number of open positions divided by the total budgeted positions. The latter includes both vacant and filled positions at the county level, i.e., the sum of the number of filled positions and the number of vacancies.^8^ It represents the desired number of NP all NHs would like to employ. The county represents an appropriate geographic fit for the nursing labor market as nursing is typically a local occupation, with nursing personnel reporting average commute times of less than half an hour [62].

To differentiate the personnel shortages by types of qualification, we calculate qualification-specific vacancy ratios in every county over time. We provide an equation concerning the construction of our measure of nursing shortage and the way we differentiate by qualification level along with additional details in the Appendix (see Eq. B1). Our findings are insensitive to excluding additional professions which tend to only partly engage in the supply of care such as social workers [e.g., 63], namely social pedagogic, social pedagogic, other professional degrees, and individuals in training, from the group of NA (results are available upon request).

### Estimation strategy

We employ a multiple linear regression model using pooled cross-sectional data to explore the relationship between NH metrics and NP vacancies. We focus on measuring NH metrics at the facility level, while NP vacancies are observed at the county level. We incorporate time fixed effects in our regression analyses, as well as regional fixed effects based on divisions defined by medical review boards having administrative functions in the German health care sector. The size of the area covered by each medical review board is comparable to the sixteen German federal states, but more homogeneous in size since two city states are mapped to neighboring states and the largest state, North Rhine-Westphalia, is split into two administrative units. We also include fixed effects based on the type of ownership of the facilities. These fixed effects aim to increase estimation efficiency by reducing variation in the outcome variables. For instance, these help abstracting from the tendency to a larger workforce among non-profit NHs.

The following equation formally describes the employed multiple linear regression model for NP-vacancy:

1 $$\begin{aligned} \begin{aligned}&\text {M}^{i}_{j,t}=\beta _0+\beta _1 \text {vacancy}^{i}_{c,t}+\beta _2{\textbf{X}}_{j,t}\\&+ \beta _3{\textbf{X}}_{c,t}+\tau _t+\kappa _r+\rho _p+\epsilon _{j,c,t} \end{aligned} \end{aligned}$$

where i denotes either NP, RN, or NA, and M represents different NH metrics. The outcome variables are measured at the facility level (*j*) and the NP-vacancy at the county level (*c*). We include control variables both at the facility as well as county level. Furthermore, we include time fixed effects ($\tau _t$), regional fixed effects at the medical review board level ($\kappa _r$),^9^ and ownership type fixed effects ($\rho _p$). The vector $X_{c,t}$ includes facility characteristics, working population, medical infrastructure, wealth, and demographics.

Aimed at mitigating the risk that our observed relationships between vacancy ratios and the NH metrics are driven by other factors, we employ control variables that might influence NH capacities including facility characteristics, working population, medical infrastructure, wealth, and demographics [27, 39, 40, 64–66]. Standard errors are clustered at the NH level. Results are robust to clustering standard errors at the county level and available upon request.^10^

To rule out concerns of multicollinearity, we pairwise calculate Pearson correlations and variance inflation factors. We find no abnormalities or high variance inflation factor values, indicating that multicollinearity is not an issue in the regression analysis (results are available upon request).

## Results

In this section, we first present descriptive statistics and then report the results for the relationship between NH metrics and nurse shortage.

Table 1 presents descriptive statistics for the key variables. On average, 42 NHs are located within one county, each providing residence for, on average, 70 residents with a mean age of slightly below 83 years. The average NP-to-resident ratio in a facility is 0.42, indicating that approximately one NP-FTE supervises two to three residents. On average, a NH employs 15 RN and 13 NA (all numbers represent FTE). The RN-to-resident ratio equals 0.23, which corresponds to slightly more than five residents per RN. For assistant nurses (NA), the average ratio is 0.19, i.e., slightly lower than the ratio for RNs. This latter finding is also documented by the average skill mix. The mean NA share equals 0.45 following our broad definition. Descriptive information for further control variables including additional facility-specific and regional information as well as data on professional home care and informal care are provided in the Appendix in Table 5.

**Table 1:** Descriptive statistics

	2007–2017			
	Mean	sd	p10	p90
*Basic NH information*				
Nursing homes, county	42.21	45.40	13.00	62.00
All nurses (NP, FTE)	28.41	16.77	10.75	48.75
Registered nurses (RN, FTE)	15.16	9.04	6.00	26.25
Nursing assistants (NA, FTE)	13.25	8.89	3.750	24.00
NH residents	70.27	40.76	25.00	120.0
Age of NH residents	82.77	3.810	79.12	86.07
*Personnel-to-resident ratios*				
NP (FTE)	.42	.16	.31	.53
RN (FTE)	.23	.12	.16	.31
NA (FTE)	.19	.08	.11	.27
*Skill-mix*				
NA share	.45	.12	.31	.58
*Beds and residents per NH*				
Nursing home size (beds)	79.88	46.17	29.00	137.0
No. residents per NH	70.31	40.75	25.00	120.0
Occupancy ratio	.89	.13	.73	1.000
*Vacancy ratio*				
NP-vacancy	.05	.02	.03	.08
RN-vacancy	.07	.03	.03	.10
NA-vacancy	.04	.02	.01	.07
Observations	59,865			

Data source: Long-term care (LTC) statistics (facility level), vacancies from the German Institute of Employment Research (IAB) (county level), INKAR data (county level). 59, 903 observations (nursing home LTC facilities): [six waves] 2007 (9,205), 2009 (9,676), 2011 (9,972), 2013 (10,197), 2015 (10,383), 2017 (10,432). German LTC statistic: Research center of the Federal and State Statistical Offices, care statistics, (10.21242/22411.2007.00.02.1.1.0–10.21242/22411.2017.00.02.1.1.0)

Regarding the regional variation, vacancy ratios tend to be somewhat lower in Southern Germany and, on average, higher for RNs than for NAs. Vacancy ratios for RNs are, e.g., especially high in the North and North-East of Germany, while the East shows particularly high vacancy ratios for NAs. Figure 4 in the Appendix illustrates the regional variation of NH vacancy ratios separately for all NP, RNs, and NAs aggregated at the spatial planning region level.

As the time trend of the average vacancy ratios, NP-vacancy goes down between 2007 and 2009, but has continuously increased since (compare Fig. 5 in the Appendix). While the overall development of RN- and NA-vacancy ratios is similar, the RN-vacancy ratio has experienced a small decline between 2011 and 2013 and the NA-vacancy ratio between 2009 and 2011. Overall, the vacancy ratio appears low relative to the extent of the public discourse about the nursing shortage problem, which is consistent with an underestimated nurse shortage. Still, the variation across counties is very informative since this underestimation is true for all regions. Moreover, a linear regression at the county level with county fixed effects and wave fixed effects shows that the number of advertised nursing personnel vacancies in elderly care rises when counties experience an increase in the number of care-dependent people per 10,000 inhabitants, i.e., changes in labor demand (results are available upon request).

**Fig. 1:**
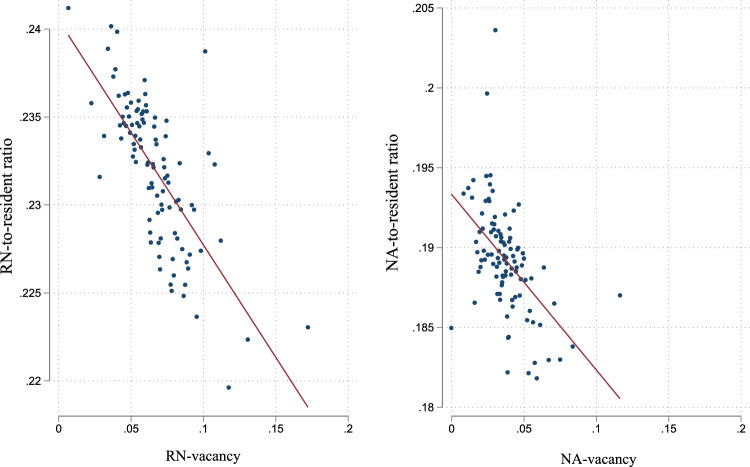
Scatter plot of nurse-to-resident ratios and nurse vacancies by qualification. *Note:* Fig. 1 is a bin scatter plot with RN left and NA right, which clusters all observations into 100 bins with comparable observations. Covariates and fixed effects are included. *Source:* (1) Care Statistic: Statistical Offices of the Länder, (10.21242/22411.2007.00.02.1.1.0–10.21242/22411.2017.00.02.1.1.0). (2) Vacancy data: Research Institute of the Federal Employment Agency, IAB

**Fig. 3:**
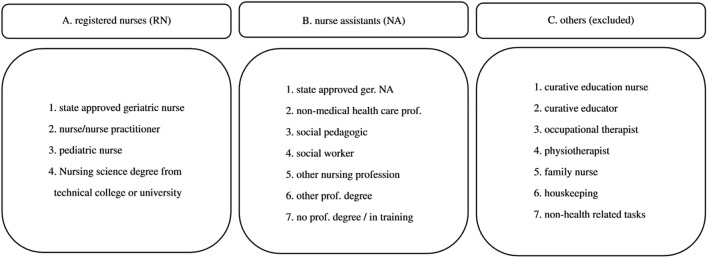
Overview of nurse qualifications. *Note: We follow the profession classification of the Research Institute of the Federal Employment Agency* ([57], *IAB).* **A.** Registered nurses (RN) are professional caregivers with a specific vocational training of 3 to 5 years. They provide basic care, medical treatments, and administrative support. **B.** Nurse assistants (NA) are caregivers with a vocational training of 1 to 2 years. They assist with personal hygiene, meal assistance, and, under supervision, administer medications and work alongside other RNs. **C.** We exclude professions that do not involve direct care contact with residents

In Fig. 1, we descriptively observe a negative linear relationship between the vacancy ratio and the nurse-to-resident ratio controlling for time-varying regional covariates.

Our regression results for the nurse-to-resident ratios are displayed in Table 2. The first column presents the results for the shortage of all NP. The findings reveal a statistically significant negative point estimate of $-0.17$. In a county with a one standard deviation higher NP-vacancy (2 percentage points), the NP-to-resident ratio is lower by 0.34 percentage points or 0.8 percent. The second and third columns display the estimates for the respective ratios for RNs ($-1.8$ percent in a county with a one standard deviation higher RN-vacancy) and NAs ($-1.2$ percent, respectively).

**Table 2:** Nurse-to-residents rates and nursing vacancies

	Nurse-to-resident ratio		
	NP	RN	NA
NP-vacancy	− .1721*** (.0345)		
RN-vacancy		−.1370*** (.0213)	
NA-vacancy			−.1117*** (.0213)
Controls	X	X	X
Wave FE	X	X	X
Regional FE	X	X	X
Owner FE	X	X	X
Mean outcome variable	0.421	0.232	0.189
Observations	59,865	59,865	59,865
F-statistics	43.05	41.29	52.87
$\hbox {R}^2$	0.052	0.060	0.045

Notes: $$^{*}\,p<0.10$$, $$^{**}\,p<0.05$$, $$^{***}\,p<0.01$$. Data source: Care statistics [56] (facility level), transparency reports (facility level), vacancies from the German Institute of Employment Research [7] (county level), indicators and maps of regional and urban development [58] (county level). 59,865 NH observations over six waves 2007–2017. Outcome: Nurse-to-resident ratio by qualification. Explanatory variable: Vacancy ratio nurses (NP-vacancy), Vacancy ratio registered nurses (RN-vacancy), Vacancy ratio nurse assistants (NA-vacancy). Control variables at the facility level and at the county level in Table 5 in the Appendix. Standard errors are clustered at the nursing home level

Since preferences for NH care, as well as regulations regarding NP requirements differ across Germany by state, we additionally analyze the vacancy ratios in the counties relative to the state average. The results presented in the Appendix are in line with those from our main specification and show that higher vacancy ratios correspond to lower state NP-to-resident ratios relative to the state average (see Table 6 in the Appendix). Hence, we can eliminate the concern that state regulations or varying long-term care policies influence our findings.

We also examine the potential substitution effects between RNs and NAs. To assess the degree to which these two professional qualifications serve as complements rather than substitutes, we look at the skill composition in terms of the share of NAs relative to RNs. Both the RN-vacancy ratio and NA-vacancy ratio are significantly correlated with the qualification mix (Table 7 in the Appendix). The share of NAs is 0.5 percentage points or 1 percent larger if the vacancy ratio for RN per county is larger by 1 standard deviation (or 0.3 percentage points). On the contrary, it is smaller if the shortage of NA increases (− 0.6 percent at the mean if NA-vacancy is 2 percent higher). Based on these results, we conclude that, to some degree, NAs act as substitutes for the shortage of RN and vice-versa. Hence, NHs have a valuable lever to manage a skill-specific nurse shortage.

**Fig. 4:**
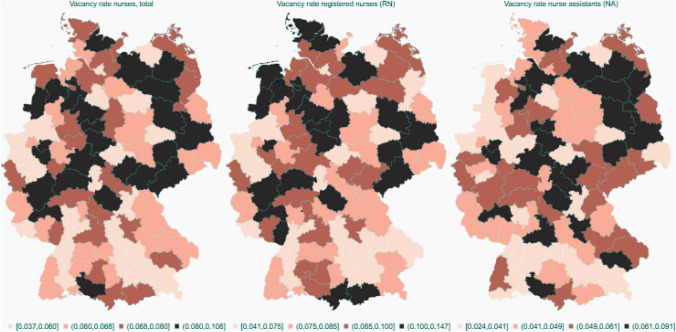
Vacancy ratios across the German spatial planning regions in 2017. *Note:* Vacancy ratios are derived from own calculations as the number of open positions divided by the total budgeted positions. We calculate the vacancy ratio for all nurses (NP), registered nurses (RN) and nursing assistants (NA). Profession definitions are provided in Fig. 3. The 97 spatial planning regions (groups of counties) correspond to OECD TL3. *Sources:* (1) Care Statistic: Statistical Offices of the Länder, (10.21242/22411.2007.00.02.1.1.0$-$10.21242/22411.2017.00.02.1.1.0) (2) Vacancy data: Research Institute of the Federal Employment Agency, IAB

**Fig. 5:**
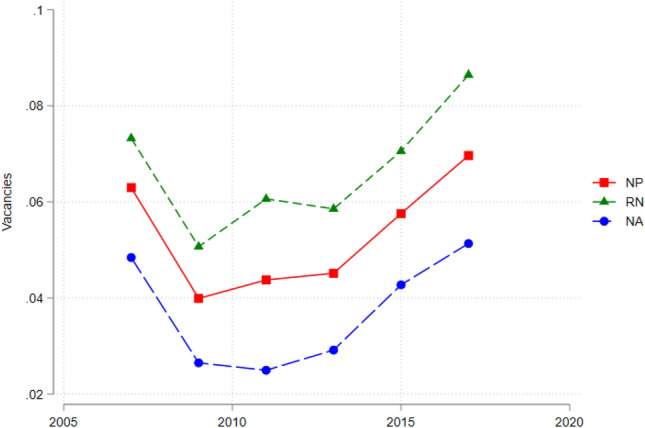
Vacancy ratios by nurse qualification from 2007 to 2017. *Note:* Vacancy ratios are derived from own calculations as the number of open positions divided by the total budgeted positions. We calculate the vacancy ratio for all nurses (NP), registered nurses (RN) and nursing assistants (NA), respectively. Profession definitions are provided in Fig. 3. *Source:* (1) Care Statistic: Statistical Offices of the Länder, (10.21242/22411.2007.00.02.1.1.0$-$10.21242/22411.2017.00.02.1.1.0) (2) Vacancy data: Research Institute of the Federal Employment Agency, IAB

Finally, we show that the occupancy rate is significantly lower with a higher vacancy ratio (compare Table 3). For instance, for a one standard deviation higher RN vacancy ratio, the occupancy rate is 0.32 percentage points lower (or − 0.4 percent at the mean of 89 percent). The effect is slightly smaller for NP-vacancy (− 0.27 percentage points) and less pronounced for NA vacancy (− 0.2 percentage points). Considering a difference in the RN-vacancy ratio between several adjacent spatial planning regions of 10 percentage points (Fig. 4 in the Appendix), our estimation results imply a difference in the occupancy rate between the same planning regions of roughly 1.2 percentage points.

**Table 3:** Occupancy rates and vacancy rates

	Occupancy rate		
NP-vacancy	−.1212*** (.0276)		
RN-vacancy		−.1057*** (.0217)	
NA-vacancy			−.0900*** (.0304)
Controls	X	X	X
Wave FE	X	X	X
Regional FE	X	X	X
Owner FE	X	X	X
Mean outcome variable	0.886	0.886	0.886
Observations	59,865	59,865	59,865
F-statistics	124.90	125.05	124.74
$\hbox {R}^2$	0.175	0.175	0.175

$$^{*}\,p<0.10$$, $$^{**}\,p<0.05$$, $$^{***}\,p<0.01$$. Data source: Care statistics [56] (facility level), transparency reports (facility level), vacancies from the German Institute of Employment Research [7] (county level), indicators and maps of regional and urban development [58] (county level). 59,865 NH observations over six waves 2007–2017. Outcome: Nursing home specific occupancy rate (i.e., occupied beds per available long-term beds). Explanatory variable: Vacancy ratio nurses (NP-vacancy), Vacancy ratio registered nurses (RN-vacancy), Vacancy ratio nurse assistants (NA-vacancy). Control variables at the facility level and at the county level in Table 5 in the Appendix. Standard errors are clustered at the nursing home level

As the occupancy rate is a function of the number of residents and the number of beds in a NH, we separately analyze the two metrics, i.e., we look at the number of residents per NH and beds per NH (Table 8 in the Appendix). While our findings do not show any significant relationships between vacancy ratios and the average number of NH beds, we observe that the NH has 1.4 fewer residents considering the distance of the $10^{th}$ percentile of the NP-vacancy ratio to the $90^{th}$ percentile (0.03−0.08 percent). The results for RN are, in absolute terms, slightly smaller but statistically different from zero. On the contrary, those for NA are statistically insignificant.

These findings appear plausible. While altering the number of beds is arguably difficult in the short and even medium term, due to regulated nurse-to-resident ratios, the number of residents and, thus, the occupancy rate is directly related to personnel shortage and especially to a shortage of RN. Since NHs need a high occupancy rate to generate sufficient income, this finding suggests an increase in the risk of NHs at the margin of exiting the care market when the nursing shortage increases.

## Discussion

This paper finds that nurse-to-resident ratios decrease as vacancy ratios increase. It also demonstrates how the qualification mix of the nursing workforce correlates with the labor shortage of RN and NA. Additionally, it documents a moderate reduction in occupancy rates driven by fewer residents. While this study is the first to empirically examine the impact of nursing personnel shortages on long-term care in nursing homes, particularly within the context of legally mandated nurse-to-resident ratios, its findings speak to those of Ching et al. [25]. Like in the present paper, the authors also link stricter nursing care regulations to reduced access. Albeit results for the hospital industry are not perfectly comparable due to differences in tasks and care needs, the results for hospital care reported by Blegen et al. [39] similarly show a negative relationship between reduced nursing supply and hours of care per care recipient.

While our paper adds important insight into how NHs relate to NP shortage, we would like to point out several potential limitations. First, multiple hires for a single vacancy or institutions not advertising vacancies as required could cause us to substantially underestimate the correlations reported in the paper. Finding nevertheless significant correlations underscores the relevance of the uncovered relationships.

Second, while our results can strictly only be interpreted as correlations, we argue that a causal effect is probable. We include various control variables to capture variation in the NH residents, substitution on the demand side, and factors that may shift labor supply more generally. We also include wave, regional, and ownership type fixed effects, effectively excluding additional potential confounding factors. Additionally, we conduct a robustness check using lagged values of our nursing shortage measure to mitigate concerns of underestimated point estimates and the presence of dynamic effects when, for instance, the implementation of NH decisions in reaction to nursing shortages take some time to implement. The results are qualitatively similar. Our results are unlikely to be confounded by changes in the case mix over time, as the baseline specification accounts for the weighted average care level of nursing home residents in each county, as well as the county-level proportion of individuals with care level 3 or higher receiving home health care or informal nursing care. Moreover, our findings remain qualitatively unchanged when controlling for the county-level share of people with care level 3 or higher in NHs. Results for the various robustness tests are available upon request.

Third, several policy changes have been implemented in Germany over time that are only partially covered by our observation period. For instance, reimbursement payments to NHs for NP increased and the link between reimbursement, minimum staffing ratios within qualification levels, and the care level mix of the residents was strengthened toward the end of the observation period. These changes are therefore insufficiently reflected in our estimation results, which could pose challenges when applying them to the current situation. For instance, an increasingly stricter regulation of staffing ratios could weaken the observed negative relationship between nursing shortages and the nurse-to-resident ratio, while yielding a stronger association between nursing shortages and the occupancy ratio. Along the lines of Winter et al. [40], this holds if there are NHs for whom the stricter regulations are binding, i.e., staffing levels are close to the mandated ratios. However, this assumption may be questioned if nursing homes can increase wages in response to nursing shortages and, according to theory, expand their nursing workforce (Section 2). For instance, the introduction of the opportunity to negotiate additional funding for NP as part of the latest nursing care reform may allow NHs to pay higher wages.^11^

We believe our findings remain highly relevant for current health policy for several reasons. For one, we carefully describe the fundamental regulatory constraints that NHs faced throughout the intervention period. While the regulatory framework differs in its particularities across countries, staffing regulations, for instance, are a widely used method to guarantee nursing care standards [67]. For another, the relevance of our study is likely to hold despite changes of the regulatory framework over time within a country. In fact, nursing shortages have remained high in Germany. In the case of NAs, shortages have even intensified [68].^12^

In the light of our findings, we now reflect on existing evidence to inform policymakers who aim to mitigate the impact of a disproportionate number of retiring workers resulting from the demographic shift. Consistent with our theoretical considerations, it seems crucial for them to facilitate that competitive wages can be offered and working conditions improved. In fact, increased wages can positively affect the enrollment in nursing programs [e.g. 22]. This may require more flexible pricing models. These can, for instance, put less emphasis on high occupancy rates to reduce financial pressures resulting from nursing shortages. More generally, public programs aimed at enhancing job stability as well as benefits can be effective in encouraging young individuals to pursue nursing careers [e.g. 24], but such policies should be part of a holistic plan for achieving improved staffing levels [20]. Potential measures to lessen the workload of nurse practitioners include streamlined access to electronic health records, the implementation of telemedicine for remote consultations, the use of wearable technology for continuous patient monitoring, and the deployment of assistive robotic systems in NHs and home healthcare settings.

Tomblin Murphy et al. [21] simulate the effects of various policies to balance the supply and demand for RN. Their findings indicate that policymakers following an integrated approach, that focuses on attracting new nursing staff, retaining existing nurses, reducing absenteeism, and enhancing productivity, can reduce nursing shortages within a few years. A care competence act is currently drafted that aims at enlarging the competencies of nurses in Germany and increasing the incentives to invest into nursing education and a career in nursing.

## Conclusion

This study explores the relationship between NP shortage and long-term care services of NHs, a topic of increasing relevance given the demographic development in most high-income countries. We measure personnel shortage using county-level vacancy ratios, differentiated by the skill-level of NP. We observe a negative correlation of the vacancy ratios with the nurse-to-resident ratios. We also show an association with the qualification mix of the nursing workforce, depending on the degree of shortage for different types of nurses. We further find a moderate reduction in the occupancy rate of NHs. This finding is supported by the statistically insignificant relationship between nursing shortages and the number of NH beds, which indicates that managing the shortage of nurses by reducing the number of beds may not be an immediate solution.

Our study sheds light on the expected changes in the NH market as a result of increasing personnel shortage. For one, the number of residents per nurse is expected to increase while the individual time for each resident and therefore the intensity of care is expected to decrease. The resulting time pressure for nurses is likely to compromise patient safety, reduce the attractiveness of the profession, and increase the risk of mistreatment of NH residents. For another, while NH size does not correlate with nurse shortages in our study, occupancy rates and the number of residents are lower when vacancy ratios are higher. A persistently reduced occupancy rate can imply the financial instability of NHs, especially if these are already at an elevated risk to exit the market [18]. Substantial anecdotal evidence corroborates the relationship between nursing home closures and financial losses stemming from staffing shortages [e.g., 70, 71]. This observed pattern warrants further empirical investigation to quantify the economic impact, e.g., by employing a longer time frame to empirically analyze the relationship between nursing shortages and NH closures. Future research could also explore effect heterogeneity across state-specific regulations.

## Figures and Tables

**Fig. 2:**
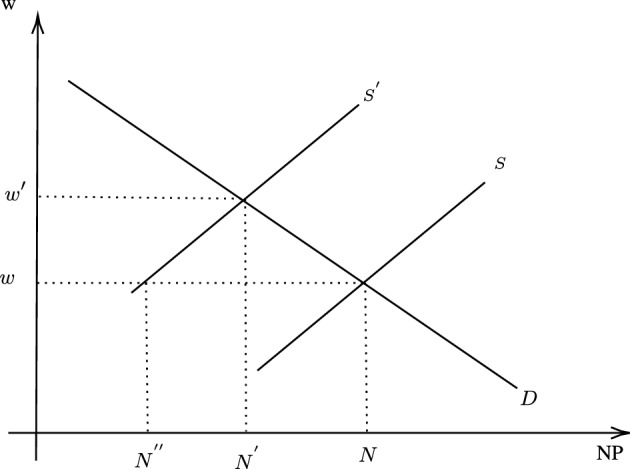
Illustration of the nurse labor market in case of personnel shortages

## Data Availability

The German Care Statistic data was obtained via the following DOI-Number references: 10.21242/22411.2007.00.02.1.1.0 (2007) and 10.21242/22411.2017.00.02.1.1.0 (2017) at the following URL: https://www.forschungsdatenzentrum.de/de/10-21242-22411-2007-00-02-1-1-0. The information on open nursing positions had been made available by the Statistik der Bundesagentur für Arbeit (Research Institute of the Federal Employment Agency: https://statistik.arbeitsagentur.de). The INKAR dataset, containing regional demographic and economic data for Germany, was freely accessible at https://www.inkar.de.

## Appendix A: Theoretical considerations

We apply a standard equilibrium model of the labor market to explain optimal demand of NHs on the nursing labor market. Assuming that fewer nurses are available, we postulate a shift in the labor supply curve, which indicates that, at a specific wage, fewer NP is willing to provide care. This causes the equilibrium between demand and supply of labor to be reached at a higher wage and a reduced number of nurses who provide care in the market.

Appendix Figure 2 shows this relationship between nursing personnel, NP, (x-axis) and wages (y-axis). The nursing supply at a given wage is displayed by *S*. The demand for nurses is displayed by *D*. A general equilibrium exists with *N* and *w*. For an illustration of the implications of nursing shortages, we introduce a shift of the NP supply curve from *S* to $S^{'}$, which results in a different equilibrium at $N^{'}$ and $w^{'}$ with less nurses at at higher wage. The shift can be interpreted as regional differences.

While wages have increased over time in Germany, the available evidence indicates a moderate sensitivity of the NH prices to local conditions [e.g., 32, 51], imposing indirect bounds on what NHs can afford to pay their NP. This is explained by difficult and time-consuming negotiations about the NH reimbursement rates between the providers and the long-term care insurers that have provided NHs limited margins to pay higher wages [52, 53]. To illustrate the implications of this plausible rigidity in the labor market, we now assume an extreme case where wages cannot increase at all. In this extreme case of full wage rigidity, we observe a reduction from *N* to $N^{''}$, i.e., even less nurses supplying care in the market when there is a shift of the NP supply curve.

Consistent with the focus of the empirical analyses that look at the shift on the supply side, we abstract from shifts of the demand curve resulting from the demographic change. With flexible wages, an increased demand for nursing care may offset the effects of nurse supply shortages as the willingness to pay for nursing services will provide incentives for individuals to supply more labor in the nursing market. Under this condition, it will be an empirical question whether the supply- or demand-side effect of the demographic shift prevails. Yet, in the extreme scenario of wage rigidity, changes on the demand side are not affecting the effects of a shift in the supply of nurses.

Note that besides wages, non-pecuniary aspects also determine the market equilibrium [72]. Consistent with the work of Kroczek and Späth (2022) [34], these factors significantly influence labor supply decisions by affecting the wage elasticity of labor supply. These can be incorporated into the model by using a relatively steep supply curve, indicating that only substantial wage increases will cause nurses to supply more labor (Appendix Table 4).

**Table 4:** Nurse-to-resident regulation in December 2017

	Care level 1	Care level 2	Care level 3	Care level 4	Care level 5
Baden-Wuerttemberg	6.11−4.47	4.76−3.49	3.26−2.47	2.55−1.90	2.32−1.72
Bavaria	6.70	3.71	2.60	1.98	1.79
Berlin	7.25	3.90	2.80	2.20	1.80
Brandenburg	4.21	3.28	2.89	2.25	1.76
Hamburg	13.40	4.60	2.80	1.99	1.77
Hessen	3,9 for care level 2 with equivalence numbers for other care levels				
Lower $\hbox {Saxony}^{*}$	6.50−4.60	4.29−3.70	3.00−2.59	2.27−1.96	2.05−1.76
Mecklenburg	8.05−6.08	4.52−3.59	3.41−2.40	2.71−1.76	2.48−1.76
Western-$$\hbox {Pommerania}^{**}$$					
North-Rhine Westphalia	8.00	4,66	3,05	2,24	2,00
Rhineland-$$\hbox {Palatine}^{***}$$	7.00	4.07	3.23	2.56	1.80
Schleswig-Holstein	6.96−5.71	5.43−4.46	3.99−3.28	3.12−2.56	2.81−2.31
Saxony-Anhalt	−	3.67−4.50	2.70−3.34	2.11−2.61	1.82−2.10

Regulation at the state level: Number of residents per registered nurse (full-time equivalents). There are no regulations in Bremen, Saarland, Saxony, and Thuringia. $$^{*}$$The regulation took place on April 1st, 2019. At the end of 2017 nursing homes had various option to translate the requirements from the old care levels (Pflegestufe) to the new care levels (Pflegegrad) [55]. Source: [54]; $$^{**}$$Values based on [73]. $$^{***}$$Values based on [74]

## Appendix B: Main explanatory variable

To differentiate the personnel shortages by types of qualification, we calculate qualification-specific vacancy ratios for all NP, RN, and NA for each qualification level *i* in county *c* at time *t* using the following equation:

B1 $$\begin{aligned} & \text {vacancy}^{i}_{c,t}=\frac{vac^{i}_{c,t}}{bud.\; pos.^{i}_{c,t}}=\frac{vac^{i}_{c,t}}{filled \; pos.^{i}_{c,t}+vac^{i}_{c,t}},\; \nonumber \end{aligned}$$

where i denotes either NP, RN, or NA. The variables $vac^{i}_{c,t}$ and ${bud. pos.^{i}_{c,t}}$ capture the number of vacancies and budgeted positions, respectively (Appendix Figs. 3, 4 and 5).

## Appendix C: Data and results

We display below Appendix Tables 5, 6, 7 and 8.

**Table 5:** Descriptive statistics for the control variables

	2007-2017			
	Mean	S.D	p10	p90
*Facility characteristics*				
Share of single-rooms	.62	.28	.21	1.00
Weighted average price, NH care [EUR]***	1,649	437	1,104	2,200
Age stationary LTC recipients	82.78	3.820	79.13	86.08
Share nonprofit	.54	.50	.00	1.00
Share private	.41	.49	.00	1.00
Share public	.05	.22	.00	1.00
*Working population*				
Female unemployed ratio	.07	.03	.03	.12
Share female unemployed older 55	.18	.05	.12	.25
Share unemployed professionals	.42	.06	.35	.50
Share employed in service sector	.22	.04	.17	.28
*Medical infrastructure*				
GPs [/100,000 inh.]	201.3	49.56	147.0	251.7
Hospital beds [/10,000 inh.]	6.09	3.05	2.79	9.70
Amb. care facilities per county	59.00	93.81	13.00	99.00
Ambulatory personnel [/10,000 inh.]	24.97	7.310	16.65	35.23
*Regional wealth*				
Monthly household income [EUR]	1,677	232	1,393	1,963
Monthly gross income [EUR/inhabitant]	2,398	396.3	1,916	2,902
Communal debts [EUR/ inhabitant]	1,581	1,320	364.6	3,237
Land price [EUR/$m^2$]	154.5	180.2	31.70	327.0
Avg. pension [EUR]	844.8	80.79	735.5	952.5
Share in need of social assistance	.22	.15	.071	.45
*Demographics*				
Share age group 50**–**65	.21	.02	.18	.25
Avg. age female population	45.05	2.01	42.80	48.10
Population density [/$km^2$]	694.4	932.5	95.00	2,113
Rurality [% inh. in region $\le 150\, inh./ km^2$]	.25	.27	.00	.68
Care-dependency, weighted average	1.77	.21	1.53	2.02
Share severe need of amb. LTC recipients	.13	.05	.08	.21
Share severe need of inf. LTC recipients	.09	.03	.06	.14
Ppl in need of inf. LTC per county	6,236	9,570	1,513	10,773
Observations	59,865			

The variables are used as covariates in the regression model to account for factors potentially influencing the capacity of NHs. For instance, the demographic variables collectively approximate the overall care level needs of the population in the county where the nursing homes are located. They aim to capture staffing effects stemming from long-term care demand rather than staff shortages. Ambulatory care personnel and facilities control for developments in the professional home health care market [75]. Data source: Care statistics [56] (facility level), vacancies from the German Institute of Employment Research [7] (county level), INKAR data [58] (district level). 59,903 observations (nursing home LTC facilities): [six waves] 2007 (9,211 obs.), 2009 (9,678 obs.), 2011 (9,973 obs.), 2013 (10,200 obs.), 2015 (10,389 obs.), 2017 (10,452 obs.). German LTC statistic: Research center of the Federal and State Statistical Offices, care statistics, (10.21242/22411.2007.00.02.1.1.0–10.21242/22411.2017.00.02.1.1.0)

**Table 6:** Absolute difference between nurse-resident rate in nursing home to federal state average and vacancy rates

	NP-to-resident diff	NR-to-resident diff	NA-to-resident diff
NP-vacancy	−.1440*** (.0345)		
RN-vacancy		−.1200*** (.0213)	
NA-vacancy			−.1010*** (.0212)
Controls	X	X	X
Wave FE	X	X	X
Regional FE	X	X	X
Owner FE	X	X	X
Mean outcome variable	0.000	0.000	0.000
Observations	59,865	59,865	59,865
F-statistics	8.28	10.13	4.73
$R^{2}$	0.025	0.033	0.008

$$^{*}\,p<0.10$$, $$^{**}\,p<0.05$$, $$^{***}\,p<0.01$$. Data source: Care Statistics [56] (facility level), transparency reports (facility level), vacancies from the German Institute of Employment Research [7] (county level), indicators and maps of regional and urban development [58] (county level). 59,865 NH observations over six waves 2007–2017. Outcome: Difference between nursing home specific nurse to resident ratio and federal state average. Explanatory variable: Vacancy ratio nurses (NP-vacancy), Vacancy ratio registered nurses (RN-vacancy), Vacancy ratio nurse assistants (NA-vacancy). Control variables at the facility level and at the county level in Table 5 in the Appendix. Standard errors are clustered at the nursing home level

**Table 7:** Qualification mix and nursing vacancies

	Share NA to RN	
RN-vacancy	.1527*** (.0232)	
NA-vacancy		− .1292*** (.0309)
Controls	X	X
Wave FE	X	X
Regional FE	X	X
Owner FE	X	X
Mean outcome variable	0.450	0.450
Observations	59,865	59,865
F-statistics	84.70	83.76
$\hbox {R}^2$	0.081	0.081

$$^{*}\,p<0.10$$, $$^{**}\,p<0.05$$, $$^{***}\,p<0.01$$. Data source: Care statistics [56] (facility level), transparency reports (facility level), vacancies from the German Institute of Employment Research [7] (county level), indicators and maps of regional and urban development [58] (county level). 59,865 NH observations over six waves 2007–2017. Outcome: Share of nurse assistants to registered nursed (qualification mix) in NH. Explanatory variable: Vacancy ratio nurses (NP-vacancy), Vacancy ratio registered nurses (RN-vacancy), Vacancy ratio nurse assistants (NA-vacancy). Control variables at the facility level and at the county level in Table 5 in the Appendix. Standard errors are clustered at the nursing home level

**Table 8:** Access and nursing vacancies

	Permanent beds			Residents		
NP-vacancy	− 18.06 (13.36)			− 27.49** (11.55)		
RN-vacancy		− 12.18 (10.44)			− 20.56**	
NA-vacancy			− 6.261 (13.81)			− 14.13 (12.09)
Controls	X	X	X	X	X	X
Wave FE	X	X	X	X	X	X
Regional FE	X	X	X	X	X	X
Owner FE	X	X	X	X	X	X
Mean outcome variable	79.876	79.876	79.876	70.307	70.307	70.307
Observations	59,865	59,865	59,865	59,865	59,865	59,865
F-statistic	44.75	44.76	44.77	54.55	54.57	54.56
$R^2$	0.125	0.125	0.125	0.134	0.134	0.134

$$^{*}\,p<0.10$$, $$^{**}\,p<0.05$$, $$^{***}\,p<0.01$$; Standard errors clustered at the county level in parentheses. Data source: Care statistics [56] (facility level), transparency reports (facility level), vacancies from the German Institute of Employment Research [7] (county level), indicators and maps of regional and urban development [58] (county level). Observations (stat. nursing facilities): [six waves] 2007 (9,205), 2009 (9,676), 2011 (9,972), 2013 (10,197), 2015 (10,383), 2017 (10,432). Outcome: Permanent beds. Explanatory variable: Vacancy rate all nurses (NP-vacancy), Vacancy rate registered nurses (RN-vacancy), Vacancy rate nurse assistants (NA-vacancy). Control variables at the facility level and at the county level in Table 5 in the Appendix. Standard errors are clustered at the nursing home level

## Footnotes

1 Several studies show that quality of care is larger in nursing homes with a larger ratio of registered nurses and with more nurses per resident, reviewed in [8].

2 Since 2019, there is a unified nurse education system that has linked these two labor markets and facilitated substitution. However, both markets still contend with minimum nurse-to-patient ratios and ongoing nurse shortages.

3 In theory, the reduction in the short term is even more pronounced when NHs have limited margins to pay higher wages due to negotiations about the NH reimbursement rates with the payers [51–53].

4 As care is an important but not the primary input factor, hospitals may have the possibility to more flexibly adjust how care is organized relative to NHs. However, the nurse-to-patient ratio does not alter with nursing vacancies in German hospitals. This may be explained by their possibility to react to nursing shortages by changing the nature of the health services provided. Mandated staffing ratios, on the contrary, are no explanation here as these were implemented in Germany only after the study‘s observation period [40].

5 Nursing staff tend to care for all residents in a NH, irrespective of whether they are permanent or short-term residents. However, since there are, on average, only 0.69 short-term beds out of a total of roughly 80 beds per NH, we consider this imprecision in our analysis to be of minor relevance. Furthermore, including the number of short-term beds as control variable does not alter the results (estimation results upon request).

6 A separate issue is how we interpret our point estimates. For example, if the actual number of vacancies was in our data and we were able to incorporate this information into our analysis, the resulting point estimates might be smaller (larger) depending on whether the actual number of vacancies tends to be larger (smaller) than the number of advertised vacancies. However, in this case, the standard deviation in the explanatory variable would also be larger (smaller). Therefore, we tend to interpret our point estimates in terms of the standard deviation or other distributional statistics in the explanatory variable, rather than in absolute units.

7 By focusing on full-time equivalent staff, our results also capture possible effects driven by a rising number of nurses working part time.

8 This implies that aforementioned concerns in relation to measurement error of the number of vacancies applies to both the numerator and denominator.

9 We abstain from including nursing home fixed effects because the identifier for nursing homes in our data changes for a significant portion of our sample over time, which greatly reduces the available temporal variation.

10 Except of the result for the estimated relationship between the number of residents and the NP-vacancy, which turns statistically significant at the 10 percent level. Respective results for the NA-vacancy and RN-vacancy turn statistically insignificant.

11 Starting in 2020, separate nursing training programs were consolidated into a unified generalist training, abolishing fees and introducing compensation to increase its attractiveness. Moreover, in July 2023, regulators introduced a nationwide skill-mix oriented staffing regulation for NHs (Sect. 113c SGB XI). As a result, NHs may negotiate higher prices arguing that they employ more NP, although they are not required to do so. To facilitate the orientation towards a more granulated skill-mix, the strict requirements for RN-ratios are currently being revised. These requirements will likely reduce the flexibility of nursing homes in optimizing care service planning for residents with different care needs, making staffing ratios even more consequential.

12 The persistence in the nursing shortage problem, even though some of the reforms explicitly have aimed at addressing staffing problems, does not necessarily imply that these measures were ineffective given the strong upward trend fueled by the demographic shift. The coronavirus pandemic also exacerbated the nurse shortage by decreasing the labor force participation among older nurses [e.g., 69].
